# Superparasitism in the Fruit Fly Parasitoid *Diachasmimorpha longicaudata* (Hymenoptera: Braconidae) and the Implications for Mass Rearing and Augmentative Release 

**DOI:** 10.3390/insects3040900

**Published:** 2012-09-25

**Authors:** Pablo Montoya, Gabriela Pérez-Lachaud, Pablo Liedo

**Affiliations:** 1 Programa Moscafrut SAGARPA-IICA, Camino a los Cacahotales S/N, CP 30680, Metapa de Domínguez, Chiapas, Mexico; 2 El Colegio de la Frontera Sur, ECOSUR, Av. Centenario Km 5.5, CP 77014, Chetumal, Quintana Roo, Mexico; E-Mail: igperez@ecosur.mx; 3 El Colegio de la Frontera Sur, ECOSUR, Carretera Antiguo Aeropuerto Km 2.5, CP 30700, Tapachula, Chiapas, Mexico; E-Mail: pliedo@ecosur.mx

**Keywords:** biological control, Mexican fruit fly, *Anastrepha ludens*, larvae

## Abstract

Superparasitism, a strategy in which a female lays eggs in/on a previously parasitized host, was attributed in the past to the inability of females to discriminate between parasitized and non-parasitized hosts. However, superparasitism is now accepted as an adaptive strategy under specific conditions. In fruit fly parasitoids, superparasitism has mainly been studied as concerns the new association between *Diachasmimorpha longicaudata* (Ashmead) (Hymenoptera: Braconidae) and the Mexican fruit fly *Anastrepha ludens* (Loew) (Diptera: Tephritidae), wherein this phenomenon is a common occurrence in both mass rearing and field conditions. Studies of this species have shown that moderate levels of superparasitism result in a female-biased sex ratio and that both massreared and wild females superparasitize their hosts without detrimental effects on offspring demographic parameters, including longevity and fecundity. These studies suggest that superparasitism in this species is advantageous. In this paper, we review superparasitism in *D. longicaudata*, discuss these findings in the context of mass rearing and field releases and address the possible implications of superparasitism in programs employing augmentative releases of parasitoids for the control of fruit fly pests.

## 1. Introduction

Most parasitoids are able to recognise and reject hosts that were previously parasitized by conspecifics or by themselves. Although previously parasitized hosts are considered to be of lower quality for oviposition than unparasitized hosts [1], females often lay a second egg (solitary parasitoids) or a second clutch of eggs (gregarious parasitoids) in or on parasitized hosts; an act called superparasitism [2,3]. In the past, superparasitism was attributed to the inability of females to discriminate between parasitized and non-parasitized hosts and was interpreted to be the result of an error by the ovipositing female. However, although the expected fitness gains per host is lower when females superparasitize, several authors have stated that under specific conditions, superparasitism might be an adaptive strategy [2,3,4,5], which is the result of a balance between the benefits and costs of laying an egg in an already parasitized host. 

Models of superparasitism as an adaptive strategy in solitary species are based on the assumption that superparasitism has no fitness consequence for the surviving larvae (*i.e.*, it does not increase the duration of larval development or reduce the adult size) [6]. For example, convincing evidence that *Leptopilina heterotoma* (Thomson) (Hymenoptera: Eucoilidae) adults emerging from single parasitized hosts are larger than adults emerging from superparasitized hosts has not been found [7]. A report [8] stated that in *Microctonus vittatae* Muesebeck (Hymenoptera: Braconidae), larvae take longer to develop in superparasitized hosts than in single parasitized hosts, but the number of eggs per host was not recorded. As survival probability decreases with age, parasitoids should become less selective and accept more host types for oviposition; this supposition leads to the prediction that older wasps will superparasitize and accept less suitable hosts than younger ones [9], a prediction that has been supported empirically [10,11].

The conditions that are predicted to favour the evolution of superparasitism are the following: (1) when the costs of extra eggs or extra time to superparasitize are low [4]; (2) when high quality hosts are rare or the risk of adult parasitoid mortality is high [1]; (3) when there are many potential benefits, for example, when the presence of two or more eggs in one host increases the offspring survival probability by overcoming the host immune defences (*i.e.*, the insurance egg) [2,4,5]; (4) when competing conspecific parasitoids are present and might also oviposit in the same host [4,5,12]; (5) when supernumerary eggs have a lower probability of being killed by other ovipositing females (ovicide) [2,4,13]; (6) when there is an increased probability that the superparasitized hosts are rejected by subsequent conspecific females, which protects subsequent offspring from further competition [2,4]; and/or (7) when there is an increase in success from competition [2,4,13,14]. 

The benefits of self-superparasitism (*i.e.*, superparasitism performed by the same female) could increase with the risk of conspecific superparasitism [15]. The advantages of superparasitism are an increased probability of producing offspring from a host and the stabilisation of host–parasitoid interactions in solitary and gregarious parasitoids [2,16]. 

In biological control situations, the decision making of parasitoids is of interest. To obtain control, parasitoids should parasitize as many different hosts as possible, as they are required to effectively decrease the number of their hosts. In the case of fruit fly parasitoids, some evidence of superparasitism by females of several species are scattered in the literature, but superparasitism has been studied mainly in the context of the new association between the Mexican fruit fly, *Anastrepha ludens* (Loew) (Diptera: Tephritidae) and *Diachasmimorpha longicaudata* (Ashmead) (Hymenoptera: Braconidae), which has been introduced in Mexico. This behaviour is a common occurrence in mass rearing and under field conditions [5,17,18]. In this review, we synthesise the main findings related to superparasitism in this new association and discuss the possible implications for control programs aimed at managing fruit fly pests through the augmentative release of parasitoids. 

## 2. Superparasitism and Biological Control

Ideally, parasitoids used as biocontrol agents are expected to be highly efficient in finding hosts and able to discriminate between parasitized and non-parasitized hosts [19,20], which avoids superparasitism and minimizes the loss of eggs, time and energy associated with searching behavior [1]. The ability to recognize hosts that are parasitized by conspecifics (host discrimination) has been documented in representatives of most major families of the parasitic Hymenoptera [21], but this ability does not necessarily lead to the avoidance of superparasitism [2]. The tendency to superparasitize hosts has been observed in several species of parasitoid wasps used in biocontrol programs. Empirical studies have shown that the consequences of superparasitism in parasitoids can vary among species. In solitary parasitoid wasps for example, the duration of immature developmental stages increased in *Microplitis croceipes *(Cresson) (Braconidae) [22] and *Venturia canescens* (Gravenhorst) (Ichneumonidae) [23] but not in *Aphidius ervi* Haliday (Braconidae) [24]. A reduction in *V. canescens* offspring size was also shown in the wasps reared from larvae subjected to superparasitism [23], but the adult wasps from superparasitized aphid hosts were larger than those from singularly parasitized hosts in *A. ervi* [24]. Similarly, when parasitic wasps exhibit superparasitism, the consequences for biocontrol programs vary according to the species. In the case of *Trichogramma *spp. (Hymenoptera: Trichogrammatidae), a high female to egg host ratio (low host density) is conducive for superparasitism but has the adverse consequences of highly male-biased offspring and low quality in the produced insects [25]. To reduce the risk of low field efficiency among the insects produced, superparasitism in *Trichogramma maidis* Pintureau & Voegelé must be avoided in mass rearing [26]. 

In contrast, in some other species, including *D. longicaudata* (see below), superparasitism has been associated with a female-biased sex ratio [17,27]. Consistently, female-biased parasitoid sex ratios might benefit biological control programs because of the resulting increases in the population growth rates of parasitoids and because males do not contribute to pest mortality [28]. Determining which factors influence the sex ratio is important for the successful rearing of parasitoids for field release [28,29,30]. Indeed, when parasitic Hymenoptera are propagated for several generations in closed laboratory systems, the relative abundance of males and females commonly fluctuates [31].

### 2.1. The Case of Diachasmimorpha longicaudata

*Diachasmimorpha longicaudata* is a solitary larval-pupal, fruit fly endoparasitoid that is commonly used worldwide as a biological control agent [32,33]. This species is mass reared in Mexico and released in specific zones with high densities of host plants, which have been identified as reservoirs of *Anastrepha* spp. fruit fly populations (Diptera: Tephritidae) [34]. It has also been released for the control of *Ceratitis capitata *(Wiedemann) (Diptera: Tephritidae) outbreaks in Mexico [35] and massreared in Florida for the control of *Anastrepha suspensa* (Loew) (Diptera: Tephritidae) [36]. Native to the Indo-Philippine region where it attacks *Bactrocera* spp. (Diptera: Tephritidae) [37], this braconid is now established in most countries where it has been introduced [38]. Unlike some other tephritid-attacking opiines, *D. longicaudata *females forage both on the canopy and at the ground level in fallen rotten fruits [39,40,41]. Female-lifespan offspring production averages 213.4 ± 4.3 eggs [27]. 

In Mexico, *D. longicaudata* is mass reared on third-instar *A. ludens* larvae irradiated at 45 Gy (8 day-old) to prevent the eclosion of adult flies from any unparasitized pupae [42]. The irradiated larvae are exposed to adult parasitoids at a rate of three larvae per female parasitoid (approximately 7,900 larvae were exposed to 2,600 female wasps per cage each day; Table 1), which can vary with the percentage of adult eclosion, which fluctuates between 60%–65%. Adult parasitoids are fed with crystallized honey [43]. Five day-old mature females are exposed to hosts for a period of six days [42]. Because of female egg depletion, the duration of larval exposure to parasitoids varies during the day (Table 1). Following exposure to parasitoids, the host larvae are collected and placed in trays with vermiculite to allow pupation. Fourteen days later, the parasitized pupae are ready to be packed and sent to different destinations for field release. Prior to release, parasitoids are subjected to quality control parameters which include: (1) percentage of adult eclosion, (2) flight and (3) sex ratio. Full details of the rearing process have been described elsewhere [44,45]. 

**Table 1:** The number and frequency of *Anastrepha ludens* larvae exposed to *D*. *longicaudata* and the sex ratio of parasitoid offspring produced during mass rearing in Metapa, Chiapas, Mexico over a 16 week collection period from October to December, 2011.

*Anastrepha ludens*	*Diachasmimorpha longicaudata*			
Time of daily exposure	Number of exposed host larvae/unit	* Number of females per cage	Duration of exposure (h)	Obtained sex ratio ♀:♂
(1) 08:00	3,100	2,600	1	4:7
(2) 12:00	2,400	2,600	1	3:5
(3) 16:00	2,400	2,600	1:45	2:8

* The number of females per cage is influenced by the percent of adult eclosion and by adult mortality during the six days inside the cage.

**Figure 1:**
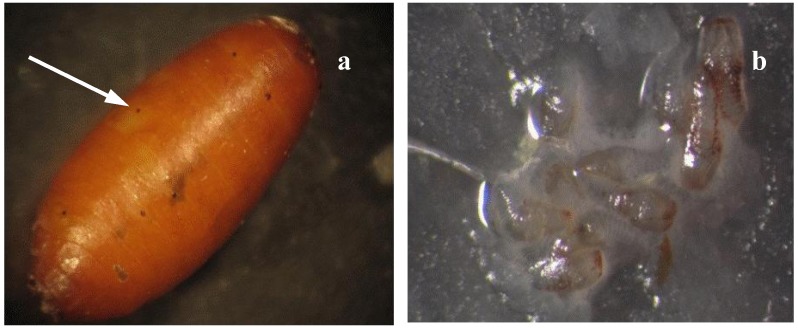
(**a**) Oviposition scars on *A. ludens* puparium, and (**b**) first instars of parasitoids observed in one dissected pupa (*i.e.*, visual evidence of superparasitism).

Superparasitism appears to be a common occurrence in *D. longicaudata*. Studies have shown that female *D. longicaudata *are able to discriminate unparasitized hosts from previously parasitized hosts [46,47], although females frequently superparasitize hosts even in the presence of high numbers of unparasitized larvae [45,47]. Routine observations at the mass-rearing facility in Mexico revealed that the puparium of over 92% of the sampled *A. ludens* pupae had multiple scars, inflicted during the last larval stage, demonstrating evidence of superparasitism (Figure 1). Previous studies have demonstrated a significant relationship between the number of oviposition scars on the puparium and the number of immatures inside the pupa [47,48]. Under these rearing conditions, more females were produced by superparasitized hosts compared to singly parasitized hosts. Superparasitism had no detrimental effects on other fitness parameters, including flight, fertility and longevity [27], which suggests that this behavior is adaptive and advantageous for biological control programs. Under laboratory conditions, parasitoids collected from wild hosts showed similar tendencies to superparasitize when compared with mass reared parasitoids [49]. Furthermore, in a choice test situation (parasitized *vs.* unparasitized hosts), 28% of mass-reared females and 30% of wild females self-superparasitized at least one host with no significant difference between female types [49]. During the five days of testing, females of both strains increased the level of superparasitism and the proportion of superparasitized hosts over time, which was interpreted as a consequence of gained experience and the physiological maturity of ovipositing females [49]. In *D. longicaudata *females, the number of mature oocytes increases as the amount of ovipositional experience increases [46]. 

Female parasitoids are capable of making sex allocation decisions which may be influenced by the previous experience of females, the host species, the host density, the host quality (*i.e.*, the size and previous parasitism) and the presence of conspecific females [29,50]. In *D. longicaudata*, the relationship between superparasitism and the female-biased sex ratio under mass-reared conditions was unexpected. The probability of an emerging parasitoid being a female was positively associated with the number of scars present on the host cuticle, which is a reliable indicator of superparasitism, but not of host size [17]. The influence of host size on sex allocation decisions of individual females seems to be overridden by superparasitism, which is positively correlated with pupa length [17]. Whether this female-biased sex ratio is the result of differential mortality between male and female larvae or of a decision by the parental female to oviposit more females in larger hosts has not been investigated. As noted in [51,52], superparasitism often yields evidence of the competitive superiority of the sex that is the best intrinsic competitor. Female parasitoids might also decide to oviposit more than one egg per host to suppress possible host defences, and the offspring gender could then be defined by internal competition [53].

The tendency of *D. longicaudata *females to superparasitize hosts has also been indicated by other studies using different fruit fly species as hosts: two eggs/larvae were observed at low host densities of *A. suspensa* in Florida, USA [46], and 20% of the *C. capitata* larvae were superparasitized in Argentina [54]. In Malaysia, *D. longicaudata *were reported to superparasitize *Bactrocera* sp. nr. *dorsalis *(Hendel) (Diptera: Tephritidae), which is a natural host of this braconid [55]. Evidence also exists that this species lays more than one egg in a multiparasitism situation: *B. dorsalis* and *C. capitata* hosts that were previously parasitized by *Fopius arisanus* (Sonan) (Hymenoptera: Braconidae) were superparasitized by *D. longicaudata *females although *F. arisanus* was found to be a superior competitor (physiological suppression) [56]. Recent studies in field populations of *D. longicaudata* in Chiapas, Mexico revealed that superparasitism is also present at high levels (~55% of parasitized pupae, but 63% of the hosts were not parasitized) and that a female-biased sex ratio was also related to this phenomenon [18]. Thus, the tendency of *D. longicaudata* females to lay more than one egg per host (*i.e.*, to superparasitize or multiparasitize hosts) appears to be a widespread characteristic of several populations or strains in the field and under laboratory conditions.

## 3. Manipulating Mass-Rearing Conditions

The conditions used to mass rear parasitoids can be manipulated to improve their sex ratios [57]. To produce more females, the mass-rearing procedures in *D. longicaudata* can be optimized through the manipulation of conditions that affect the level of superparasitism [17,27,49]. These include: (1) the ratio of host larvae to female parasitoids by increasing or diminishing the number of exposed larvae based on the sequential number of exposures during the day, and (2) the duration of larval exposure to females. As previously discussed, in the mass rearing of *D. longicaudata* the duration of host larvae exposure varies over time (Table 1), which allows the obtainment of sex ratios favorable to females. A recent study of *D. longicaudata* mass rearing [58] suggests that the duration of host larval exposure and the host density could be modified in relation to the age of females by using shorter periods of exposure for younger (5- to 7-day-old) females and longer periods for older (8- to 10-day-old) females because of a lower egg load in older wasps. Adjustment of the host density according to the females’ age is also feasible: offering more hosts to younger females in the daily exposures and fewer hosts to older ones. However, these proposals need to be evaluated under the logistics of a mass-rearing program.

There is an implicit risk in manipulating the ovipositional behavior of *D. longicaudata* females under mass-rearing conditions because it has been reported that high numbers of oviposition scars (>12) per pupa lead to high levels of host mortality and consequently low levels of adult wasp eclosion [27]. Careless management of conditions that favor superparasitism could represent a serious disadvantage by increasing the costs of mass-produced parasitoids and generating contamination problems from the opportunistic Phoridae flies associated with dead larvae [59]. 

## 4. Superparasitism in Other Fruit Fly Parasitoid Species

Superparasitism in *D. longicaudata* and other fruit fly parasitoids might be an evolutionary response to interspecific competition. In Mexico, several opiinae parasitoids form part of a guild that attacks third instar larvae of several species in the genus *Anastrepha* Schiner (Diptera: Tephritidae) [39], which includes *D. longicaudata*. Consequently, it is possible that different parasitoid species might compete extrinsically (*i.e.*, during the host selection process by adult females) and intrinsically (*i.e.*, during immature developmental stages) for access to and control of host resources (see [56]). During interspecific competition in the field, self-superparasitism of hosts might be profitable for *D. longicaudata* if the total survival rate (fitness performance) of the first and second eggs laid in selfsuperparasitized hosts is higher than that of the progeny in singly parasitized hosts. Particularly when these hosts are subsequently attacked by conspecifics or by another co-occurring parasitoid [2]; this phenomenon has been shown for *Haplogonatopus atratus* Esaki & Hashimoto (Hymenoptera: Dryinidae) under laboratory conditions [60]. Only incidental evidence of superparasitism in other fruit fly parasitoids has been published, and it is not known how widespread superparasitism is as a strategy in the guild of parasitoids that attack immature fruit flies. Possibly due to superparasitism [61], the congeneric *Diachasmimorpha kraussi* (Fullaway) appears to inflict high mortality on its rearing host *Bactrocera latifrons *(Hendel) (Diptera: Tephritidae).

Female *Coptera haywardi* (Oglobin) (Hymenoptera: Diapriidae), a pupal endoparasitoid of fruit flies, are known to show significant conspecific and heterospecific discrimination [62,63]. However, in choice tests, the females have been observed to superparasitize hosts from which only one adult emerge, but it remains unknown whether this superparasitism has an effect on fitness parameters. In a closely related species *Coptera occidentalis* Muesebeck that attacks *C. capitata*, superparasitism was frequent: 56% of the examined hosts had an average of 5.04 eggs per dissected pupa [64]. Another extensively studied species is *F. arisanus*, an egg parasitoid of fruit flies. This species has been noted to exert an impressive capacity for discrimination because a low percentage of attacked eggs (~2%) were reported as superparasitized [65,66,67]. 

A recent study [68] compared superparasitism behavior and its consequences in two mass-reared species of Opiinae parasitoids (Braconidae) that attack *A. ludens* larvae: a native species *Doryctobracon crawfordi* (Viereck) and an exotic species *Diachasmimorpha tryoni* (Cameron). The results showed that each species exhibited different foraging strategies, especially regarding superparasitism. *Doryctobracon crawfordi* did not superparasitize its hosts whether acting alone or in the presence of conspecifics, whereas *D. tryoni* exhibited superparasitism in both situations. As in *D. longicaudata* (see [17,27]), superparasitism in the congeneric *D. tryoni* did not exert any deleterious effect on survival or fecundity and was also positively correlated with a sex ratio favorable to females. 

## 5. Conclusions and Future Perspectives

This review shows that superparasitism is an ubiquitous characteristic of *D. longicaudata* populations and not a result of mass-rearing procedures as initially proposed. This trait confirms the selection of this species as a natural enemy suitable for augmentative biological control programs because a higher proportion of females is derived from superparasitism under mass-rearing conditions. This trait should contribute to improvements in the control of pest populations and compensate for the loss of individuals produced by high levels of superparasitism when managed correctly.

An area that requires future attention is the role a symbiotic virus, known to be transmitted by *D. longicaudata*, might play in suppressing host defenses and how this could benefit mass-rearing programs. *Diachasmimorpha longicaudata* females inject the virus (entomopoxvirus DlEPV) during parasitism into their hosts, which then express viral gene products that alter the host immune defenses, growth and development to optimize the conditions for the development of the wasps’ offspring [69]. 

Another area that requires consideration is the importance of superparasitism within interspecific competition in the field. Frequently, the first eggs laid by parasitoids are expected to prevail over intrinsic competition, but often survival among parasitoid larvae of the same age is found to be independent of the ovipositional sequence [70]. Furthermore, the outcome of competition might also depend on the time elapsed between the two parasitisation events [71]. 

Questions of how widespread superparasitism is in fruit fly parasitoid guilds and what the consequences are on parasitoid fitness across a range of parasitoid species remain largely unanswered. Future research on superparasitism in several fruit fly parasitoid species may further contribute to our understanding of host-parasitoid interactions and how such interactions can be manipulated to optimize the effectiveness of augmentative biological control programs.
